# Progesterone-primed ovarian stimulation in polycystic ovarian syndrome: An RCT

**DOI:** 10.18502/ijrm.v17i9.5103

**Published:** 2019-09-22

**Authors:** Maryam Eftekhar, Masrooreh Hoseini, Lida Saeed

**Affiliations:** ^1^Research and Clinical Center for Infertility, Shahid Sadoughi University of Medical Sciences Yazd Iran.; ^2^Abortion Research Center, Yazd Reproductive Science Institute, Shahid Sadoughi University of Medical Science Yazd Iran.; ^3^Afzalipour Hospital Kerman University of Medical Sciences Kerman Iran.

**Keywords:** Progesterone, Polycystic ovarian syndrome, Controlled ovarian stimulation, Frozen-thawed embryo transfer, Pregnancy rate

## Abstract

**Background:**

In vitro fertilization is an important therapy for women with polycystic ovarian syndrome (PCOS). The use of new ways of improving clinical results is yet required.

**Objective:**

This study was aimed to investigate the efficacy of progesterone primed ovarian stimulation (PPOS) and compare with conventional antagonist protocol in PCOS.

**Materials and Methods:**

A total of 120 PCOS women who were candidates for assisted reproductive technology treatment from August to January 2019 were enrolled in this RCT and were placed into two groups, randomly (n= 60/each). The PPOS group received 20 mg /day Dydrogesterone orally since the second day of the cycle and the control group received antagonist protocol. The pregnancy outcomes including the chemical and clinical pregnancy, the miscarriage rate, and the percent of gestational sacs/ transferred embryos was compared in two groups.

**Results:**

Number of MII oocyte, maturity rate, Number of 2 pronuclei (2PN) and serum estradiol levels on trigger day were statistically lower in PPOS group (p = 0.019, p = 0.035, p = 0.032, p = 0.030), respectively. Serum LH level on trigger day in PPOS group was higher than antagonist group (p = 0.005). Although there wasn’t sever ovarian hyper simulation syndrome in any participants, mild and moderate ovarian hyper simulation syndrome was less in PPOS group (p = 0.001). Also, the chemical and clinical pregnancy rate were higher in the antagonist group, althoughit was not statistically significant (p = 0.136, p = 0.093 respectively).

**Conclusion:**

Our study demonstrate that PPOS does not improve chemical and clinical pregnancy rate of the infertile women with PCOS.

## 1 Introduction

***Registration in IRCT: IRCT20110509006420N18***

Polycystic ovarian syndrome (PCOS) is a prevalent endocrine disorder. About 6.3–21.4% of women in the reproductive age suffers from this disease. In vitro fertilization (IVF) is one of the important therapy for women with PCOS. Regardless of the higher number of retrieved oocytes in PCOS patients, low fertility rate, poor oocytes quality, and high rates of abortion are yet an important issues. Therefore, new protocols are needed to improve clinical outcomes (1). Nowadays we are observing the ‘freeze-all’ techniques that freeze the entire number of oocytes or embryos, which we could utilize ovarian stimulation with no restriction, including adverse effects of hormones on endometrial receptivity (2).

It has been proved that progesterone prevents pulsatile secretion of luteinizing hormone (LH) and gonadotropin releasing hormone (GnRH) (3).

In order to inhibit LH increasing, progestin-primed ovarian stimulation (PPOS) was stated.I In this protocol oral progesterone (P) injected from the initial day of ovarian stimulation at the follicular phase (4). Using this novel PPOS protocol, P level, are utilized as the substitutions of GnRH analogue to inhibit the early LH surge during the follicular stage (5).

The effects of progestin and the freeze-all strategy suggests that progestin-primed can be used as an ideal regimen for PCOS patients who are treated with assisted reproductive technology (ART) (6). In addition, progestin administrated orally and it is more convenient.

Freeze all embryos strategy and transfer in a subsequent cycle, can reduce the late onset ovarian hyper simulation syndrome (OHSS).

To avoid hypothalamic pituitary ovarian hypoxia, gonadotropin releasing hormone agonist (GnRHa) with a low dose of hCG (1000IU) as the final triggering was used with low risk of moderate or severe OHSS. Selecting the appropriate progestin is essential for the PPOS protocol success (5).

Dydrogesterone (duphaston,) is an artificial progesterone and is highly similar to endogenous progesterone in terms of its pharmacologic properties and molecular structure. In addition, it does not interfere with endogenous progesterone production (3). This drug is widely used to treat hormone replacement, abortion, and the luteal support in pregnancy (5).

In the present study, it was hypothesized that dydrogesterone could be utilized as a substitute progestin in a PPOS protocol. We designed a randomized clinical trial (RCT) to evaluate the cycle characteristics and pregnancy outcome of individuals with PPOS and compare them with conventional antagonist.

## 2 Materials and Methods

### 2.1 Study design

A total of 120 individuals with PCOS aged between 18-40 yr old and were candidate for ART treatment were enrolled in this study. The study was taken place in Yazd Research and Clinical Center for Infertility between August to January 2019.

PCOS diagnosis was performed based on Rotterdam criteria (2003), including polycystic ovaries, oligo-anovulation, as well as the biochemical or clinical signs of hyperandrogenism (7).

Women with the history of intrauterine abnormalities (submucosal fibroma, uterine polyp, and intrauterine adhesions), severe endometriosis, systemic diseases, and azoospermia in their husbands were excluded from the study. Grouping was done by disclosing the sealed envelopes

All subjects received 150 subcutaneous doses of Cinnal-f (Cinnagen, Iran) from the 2^nd^ day of the cycle. Women in progesterone primed (PPOS) group, were prescribed 20 mg oral dose of dydrogesterone (duphaston, Abbott, Netherlands) from the 2nd day of the cycle and continued until triggering day. Vaginal sonography was done for all patients since 6th day of cycle. In the antagonist group, when the size of dominant follicles reached to 12–13 mm, 0.25 mg of cetrotide (Merck-Serono Germany) was injected subcutaneously daily and continued until triggering day.

When dominant follicles reached 17 mm, serum LH, E2 and P were checked. Then the final triggering was performed by Subcutaneous injection of decapeptyl 0.2 mg (Ferring, Germany) and intramuscular injection of human chorionic gonadotropin (HCG) 1000 IU (Pregnyl- Germany) in both groups. Oocytes pick up was done 36 hr later. All embryos were frozen in cleavage stage and frozen embryo transfer was done 2 months later.

### 2.2 Embryo vitrification and warming

Cryopreservation of all embryos was done by cryotop vitrification method on 2nd or 3rd day after oocyte retrieval in both groups (8).

### 2.3 Endometrium preparation

Endometrial preparation process were similar in both groups. All subjects received 6 mg/day estradiol valerate (Aburaihan Co., Tehran, Iran) orally from the 2nd day of menstrual cycle.

Vaginal ultrasonography was performed to measure endometrial thickness on the 13th day of menstrual cycle. When endometrial thickness reached ≥ 8 mm, all subjects received 400 mg of Cyclogest®vaginal peccaries (Cox Pharmaceuticals, Barnstaple, UK) twice a day until menstruation or 8 wk of gestational age in pregnant women. Embryo transfer was performed three days after progesterone administration.ering,. Estradiol and progesterone injection continued for up to 8 wk after pregnancy.

### 2.4 Pregnancy outcomes

Chemical pregnancy was determined by serum β hCG > 50 IU/L two wk after ET. In addition, clinical pregnancy was confirmed by detecting fetal heartbeats 2 wk following the positive β hCG. The miscarriage was defined as losing pregnancy prior to 20 wk of gestation. The implantation rate was considered as percentage of gestational sacs/ transferred embryos.

### 2.5 Ethical consideration

The Ethics Committee of Yazd Reproductive Sciences Institute approved the study protocol (code: IR.SSU.RSI.REC.1397.003). The study was registered at IRCT (Iranian Registry of Clinical Trials) under code IRCT20110509006420N18 Written informed consent was obtained from all subjects after counseling about conventional antagonist protocol and PPOS.

### 2.6 Statistical analysis

The statistical analysis was performed using SPSS software (Statistical Package for the Social Sciences, version 20.0, Chicago, IL, USA). In order of identifying major differences in the two groups, both chi-square and t-test were utilized. P < 0.05 was regarded as the significance level.

## 3 Results

**Figure 1:**
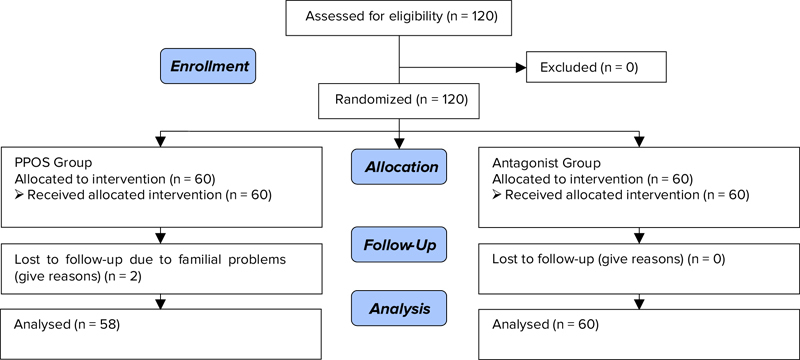
Consort Flow Diagram

A total of 120 women who met the inclusion criteria enrolled the study as 2 groups (n= 60/each). Two patients failed to follow-up in the study due to familial problems group (Figure 1). The baseline characteristics were similar in both groups (Table I).

**Table I:** Basic characteristics of the participants in two groups

Variable	PPOS group	Antagonist group	p-value
Female age (yr)^*^	28.47 ± 3.60	28.98 ± 3.55	0.433
Duration of infertility (yr)^*^	6.00 ± 2.84	6.88 ± 3.65	0.154
Type of infertility^**^			
Primary	48 (82.8)	46 (76.7)	0.411
Secondary	10 (17.2)	14 (23.3)	
AMH (IU/L)^*^	8.95 ± 3.70	9.76 ± 4.64	0.302

* Data presented as mean ± SD. student t-test,

** Data presented as n (%). Chi- square

AMH: Anti mullerian hormone PPOS: Progesterone primed ovarian stimulation Yr: Year

There was no significant different between duration of stimulation, total dose of gonadotropin and No. of retrieved oocyte between two groups. No. of MII oocyte, maturity rate, No. of 2 pronuclei (2PN) and serum E2 levels on trigger day were lower in PPOS group (p = 0.019, p = 0.035, p = 0.032, p = 0.030 respectively).

Serum LH level on trigger day in PPOS group was higher than antagonist group (5.29 vs. 3.79; p = 0.005). Although there wasn’t sever OHSS in any patient, mild and moderate OHSS was less in PPOS group (36.5% vs. 68.3%; p = 0.001) (Table II). Chemical and clinical pregnancy were more in antagonist group which was not statistically significant (Table III).

**Table II:** . Results of ovarian stimulation

Variable	PPOS group	Antagonist group	p-value
Duration of stimulation (day)^*^	10.24 ± 2.39	9.53± 2.01	0.084
Total dose of gonadotropin (IU)^*^	1528.45 ± 413.15	1430.00± 354.45	0.167
No. of retrieved oocytes^*^	15.74± 9.88	18.65 ± 7.87	0.083
No. of MII oocytes^*^	12.50± 8.88	16.03 ± 6.99	0.019
Maturity rate^***^	79.90%	85.52%	0.035
No. of two pronucleus^*^	8.70± 7.43	11.38 ± 5.68	0.032
No. of obtained embryos^*^	7.91 ± 6.63	9.48 ± 4.62	0.141
Total cycle cancelation^**^	4 (6.9%)	0 (0%)	0.038
E2 levels on trigger day (pg/mL)^*^	2351.55± 965.58	3047.68 ± 2157.04	0.030
LH levels of on trigger (IU/mL)^*^	5.29 ± 3.79	3.56 ± 2.61	0.005
P levels on trigger day (IU/mL)^*^	0.72± 1.25	0.81 ± 0.92	0.687
Fertilization rate^***^	63.26%	71.30%	0.073
Endometrial thickness (mm)^*^	9.20 ± 1.34	8.83 ± 1.14	0.161
OHSS (mild and moderate)^***^	36.5%	68.3%	0.001

* Data presented as mean ± SD. student t-test,

** Data presented as n (%). Chi- square

*** Data presented as percentages, PPOS: Progesterone primed ovarian stimulation

MII: Mature oocyte II E2: Estradiol LH: Luteinizing hormone

P level: Progesterone level OHSS: Ovarian hyper stimulation syndrome

**Table III:** . ART outcomes in frozen embryo cycles

Variable	PPOS group	Antagonist group	p-value#
Implantation rate^*^	7.32%	15.69%	0.062
Chemical pregnancy rate/transfer^**^	13 (31.7)	24 (47)	0.136
Clinical pregnancy rate/transfer^**^	6 (14.6)	15 (29.4)	0.093
Abortion rate^**^	8 (61.5)	9 (37.5)	0.161

#* Data presented as (%)

** Data presented as n (%)

Chi square PPOS: Progesterone primed ovarian stimulation

## 4 Discussion

In this present study, clinical efﬁciency of duphaston in PPOS regimen were evaluated. Our results showed that duphaston as an FSH adjuvant to during the ovarian stimulation did not lead to similar mature oocyte retrieval. Oocyte maturity was mainly utilized to assess the oocyte quality. The maturity rate of oocytes in PPOS group was significantly lower than antagonist group. In addition, fertilization rate was lower in the PPOS group.

The pregnancy results of FET in PPOS showed a lower clinical pregnancy rate, 14.6% vs. 29.9%. The implantation rate was also lower in PPOS group, although it was not statistically significant but clinically notable. Although duphaston increased the early LH rate, it did not interfere with the measurements of progesterone. However, in the present study, the mean level of LH was significantly higher in the PPOS group.

Previous studies has shown that when progesterone is administered during the regular follicular phase, it decreases the the LH pulse frequency, increases the amplitude LH pulse, and decreases the mean LH levels of plasma compared with those who were not treated (3).

The LH level reached its highest value at the mid-cycle time and causes the meiosis I reinitiate inside pre-ovulatory follicles. The mentioned time is crucial for successful fertilization, perfect embryo development, and egg maturity. It seems that there is a level of LH capacity which the overexposure can affect the prohibition of ovulation by controlling the granulosa distribution, oocyte atresia, early luteinization and ultimately affects IVF outcome. However, the appropriate level of LH on trigger day was not determined (9).

In the present study, estradiol level on trigger day was significantly lower in PPOS group.

The effect of over-physiological levels of E2 on IVF outcomes are still controversial. Some researchers have stated that the serum E2 concentrations on the day of hCG administration have a positive correlation with pregnancy outcomes. Nevertheless, some other researchers have reported adverse or no effects of high levels of E2 association between the levels of E2 serum and the IVF outcomes of (10–12). All studies that investigated the effect of estradiol on pregnancy were conducted under the fresh embryo transfer conditions (10–12). In the literature review, there was no study that made to determine the effect of estradiol serum level in trigger day on frozen embryo outcome. In the current study, the lower level of estradiol on the trigger day was correlated with the lower numbers of mature oocytes.

Kuang and colleagues conducted a primary randomized study on PPOS. They added medroxyprogesterone acetate to gonadotropin-induced stimulation in the follicular phase, and compared this protocol with traditional short protocol. They demonstrated that pregnancy, implantation, and miscarriage rates were not meaningfully different between groups (13).

In another study Nanako lwami and co-workers compared the rates of ongoing and clinical pregnancies between the antagonist regimen and the PPOS protocol. They used oral dydrogesterone and HMG in the PPOS protocol. They concluded that the rates of ongoing and clinical pregnancies were similar in both groups which is in contrast with our results. However, they included normal responders in addition to hyper-responders (14).

In a pilot study, a short protocol was compared with the PPOS protocol in PCOS patients. This article reported no significant differences in the number of collected oocyte and the incidence of ongoing pregnancy. However, a high dose of HMG was consumed in the PPOS group. Considering the particular risk of OHSS, two cases were reported in the short protocol group vs. none in the PPOS group (p = 0.154) (6).s

## 5 Conclusion

In conclusion, the results of the present study showed that PPOS is not appropriate for women with PCOS, however, the results of previous studies on PPOS were in contrast with this present study. It seems that further randomized clinical trials (RCTs) are required for better assessment of PPOS in PCOS.

## Conflict of Interest

There is no Conflict of interest

## References

[1] Zhu X, Ye H, Fu Y. (2016). The utrogestan and hMG protocol in patients with polycystic ovarian syndrome undergoing controlled ovarian hyperstimulation during IVF/ICSI treatments. Medicine.

[2] Massin N. (2017). New stimulation regimens: endogenous and exogenous progesterone use to block the LH surge during ovarian stimulation for IVF. Hum Reprod Update.

[3] Zhu X, Ye H, Fu Y. (2017). Duphaston and human menopausal gonadotropin protocol in normally ovulatory women undergoing controlled ovarian hyperstimulation during in vitro fertilization/intracytoplasmic sperm injection treatments in combination with embryo cryopreservation. Fertil Steril.

[4] Huang CY, Chen GY, Shieh ML, Li HY. (2018). An extremely patient-friendly and efficient stimulation protocol for assisted reproductive technology in normal and high responders. Reprod Biol Endocrinol.

[5] Yu S, Long H, Chang HY, Liu Y, Gao H, Zhu J (2018). New application of dydrogesterone as a part of a progestin-primed ovarian stimulation protocol for IVF: a randomized controlled trial including 516 first IVF/ICSI cycles. Hum Reprod.

[6] Wang Y, Chen Q, Wang N, Chen H, Lyu Q, Kuang Y. (2016). Controlled ovarian stimulation using medroxyprogesterone acetate and hMG in patients with polycystic ovary syndrome treated for IVF: a double-blind randomized crossover clinical trial. Medicine.

[7] Rotterdam ESHRE/ASRM-Sponsored PCOS consensus workshop group (2004). Revised 2003 consensus on diagnostic criteria and long-term health risks related to polycystic ovary syndrome (PCOS). Hum Reprod.

[8] Eftekhar M, Rahsepar M, Rahmani E. (2013). Effect of progesterone supplementation on natural frozen-thawed embryo transfer cycles: a randomized controlled trial. Int J Fertil Steril.

[9] Gardner DK, Weissman A, Howles CM, Shoham Z. (2016). Textbook of assisted reproductive techniques: laboratory and clinical perspectives.

[10] Kosmas IP, Kolibianakis EM, Devroey P. (2004). Association of estradiol levels on the day of hCG administration and pregnancy achievement in IVF: a systematic review. Hum Reprod.

[11] Serna J, Cholquevilque JL, Cela V, Martínez-Salazar J, Requena A, Garcia-Velasco JA. (2008). Estradiol supplementation during the luteal phase of IVF-ICSI patients: a randomized, controlled trial. Fertil Steril.

[12] Joo BS, Park SH, An BM, Kim KS, Moon SE, Moon HS. (2010). Serum estradiol levels during controlled ovarian hyperstimulation influence the pregnancy outcome of in vitro fertilization in a concentration-dependent manner.. Fertil Steril.

[13] Kuang Y, Chen Q, Fu Y, Wang Y, Hong Q, Lyu Q (2015). Medroxyprogesterone acetate is an effective oral alternative for preventing premature luteinizing hormone surges in women undergoing controlled ovarian hyperstimulation for in vitro fertilization. Fertil Steril.

[14] Iwami N, Kawamata M, Ozawa N, Yamamoto T, Watanabe E, Moriwaka O (2018). New trial of progestin-primed ovarian stimulation using dydrogesterone versus a typical GnRH antagonist regimen in assisted reproductive technology. Arch Gynecol Obstet.

