# Erratum to “Progesterone-primed ovarian stimulation in polycystic ovarian syndrome: An RCT” [Int J Reprod BioMed 2019; 17: 671-676

**DOI:** 10.18502/ijrm.v19i6.9380

**Published:** 2021-07-27

**Authors:** Maryam Eftekhar, Masrooreh Hoseini, Lida Saeed

The authors regret that the start date of the study was published incorrectly (Pages 671 and 672). The start date must be changed from August 2018 to May 2018. The authors would like to apologize for any inconvenience caused. The online version of article has been updated on July 14, 2021. 

